# Organic Carbon Mineralization and Bacterial Community of Active Layer Soils Response to Short-Term Warming in the Great Hing’an Mountains of Northeast China

**DOI:** 10.3389/fmicb.2021.802213

**Published:** 2021-12-24

**Authors:** Xingfeng Dong, Chao Liu, Dalong Ma, Yufei Wu, Haoran Man, Xiangwen Wu, Miao Li, Shuying Zang

**Affiliations:** ^1^Heilongjiang Province Key Laboratory of Geographical Environment Monitoring and Spatial Information Service in Cold Regions, Harbin Normal University, Harbin, China; ^2^Heilongjiang Province Collaborative Innovation Center of Cold Region Ecological Safety, Harbin, China

**Keywords:** active layer, bacterial community, carbon substrate, incubation, temperature sensitivity

## Abstract

As a buffer layer for the energy and water exchange between atmosphere and permafrost, the active layer is sensitive to climate warming. Changes in the thermal state in active layer can alter soil organic carbon (SOC) dynamics. It is critical to identify the response of soil microbial communities to warming to better predict the regional carbon cycle under the background of global warming. Here, the active layer soils collected from a wetland-forest ecotone in the continuous permafrost region of Northeastern China were incubated at 5 and 15°C for 45 days. High-throughput sequencing of the 16S rRNA gene was used to examine the response of bacterial community structure to experimental warming. A total of 4148 OTUs were identified, which followed the order 15°C > 5°C > pre-incubated. Incubation temperature, soil layer and their interaction have significant effects on bacterial alpha diversity (Chao index). Bacterial communities under different temperature were clearly distinguished. Chloroflexi, Actinobacteria, Proteobacteria, and Acidobacteria accounted for more than 80% of the community abundance at the phylum level. Warming decreased the relative abundance of Chloroflexi and Acidobacteria, while Actinobacteria and Proteobacteria exhibited increasing trend. At family level, the abundance of norank_o__norank_c__AD3 and Ktedonobacteraceae decreased significantly with the increase of temperature, while Micrococcaccac increased. In addition, the amount of SOC mineralization were positively correlated with the relative abundances of most bacterial phyla and SOC content. SOC content was positively correlated with the relative abundance of most bacterial phyla. Results indicate that the SOC content was the primary explanatory variable and driver of microbial regulation for SOC mineralization. Our results provide a new perspective for understanding the microbial mechanisms that accelerates SOC decomposition under warming conditions in the forest-wetland ecotone of permafrost region.

## Introduction

As an important link connecting the carbon cycle between the atmosphere and terrestrial ecosystems (Jobbágy and Jackson, 2000), soil organic carbon (SOC) mineralization is sensitive to changes in environmental conditions (Mu et al., 2016; Wei et al., 2017). SOC stored in permafrost regions due to their cold and wet conditions is greater than that currently within the atmosphere (Schuur et al., 2009; Tarnocai et al., 2009; Hugelius et al., 2014; Ping et al., 2015). Warming is influencing the biogeochemical cycles, and the decomposition of SOC in permafrost regions will be accelerated (Schuur et al., 2009; Ricketts et al., 2020). Moreover, SOC cycles are related to greenhouse gases which have been confirmed to be the primary contributors to global warming (Schuur et al., 2015). Therefore, warming in permafrost-influenced regions has the potential to alter the global carbon balance through feedback processes (Davidson and Janssens, 2006; Kuhry et al., 2010; Burke et al., 2013; Hugelius et al., 2014). Carbon emissions from permafrost-influenced regions have recently received increased attention under the background of climate warming.

The active layer is the soil layer overlying the permafrost, which undergoes seasonal freezing and thawing. As a buffer layer between atmosphere and permafrost, the potential changes of active layer may greatly affect biogeochemical activities. Ping et al. (2015) demonstrated that SOC mineralization in permafrost regions is mainly restricted by cold temperature. In the past few decades, high altitude and latitude regions have experienced faster climate warming compared with other regions (D’Alò et al., 2021). There is no doubt that warming will increase the depth of the active layer and reduce the temperature limit in the decomposition of organic matter, leading to elevated release of CO_2_ to the atmosphere (Rivkina et al., 2000; Schuur et al., 2009; Johnstone et al., 2010; Monteux et al., 2019). Previous studies on shallow soils have reported that increased temperature promotes SOC mineralization (Wang et al., 2010, 2014; Lang et al., 2011). Ping et al. (2008) showed that changes in the freeze-thaw environment caused by warming promote the migration of organic matter in the soil profile. The importance of decomposable SOC in the deep layers of the permafrost region has been repeatedly emphasized (Waldrop et al., 2010; Schuur et al., 2015; Mu et al., 2016; Jiang et al., 2020). The profile of active layer can be regarded as a good material to predict the changes of soil system caused by permafrost thawing (Kim et al., 2016). Therefore, the active layer has received extensive scientific attention. Determining the temperature sensitivity of SOC mineralization is of great significance for understanding the regional soil carbon process in the context of global climate warming (Wallenstein et al., 2010; Maia et al., 2019). The *Q*_10_ value represents the change in the gas emission rate under a temperature change of 10°C (Kirschbaum, 1995), and is a key indicator to explore SOC decomposition under a global warming scenario (Conant et al., 2010; Suseela and Dukes, 2013; Nottingham et al., 2016).

Soil microbes depend on organic matter such as dissolved organic carbon (DOC) to thrive regulate the turnover of substantia (Michaelson and Ping, 2003; Frey et al., 2013; Abbott et al., 2015). The functions of soil microbial communities have significant effects on the decomposition of SOC (Hultman et al., 2015; Ding et al., 2016; Ricketts et al., 2016; Walker et al., 2018). Non-psychrophilic (but cold resistant) microorganisms might have more potential than psychrophilic bacterium in obtaining organic substrates once warming reduces the temperature restrictions on its growth and activity, resulting in enhanced SOC decomposition and the release of greenhouse gases into the atmosphere, which leads to warming effect within the permafrost ecosystem (Karhu et al., 2014; Ricketts et al., 2016; Monteux et al., 2019). However, the microbiome in the southern edge of the permafrost region in Eurasia is much less understood. Hence, research on the response of the microbial community to increasing temperature is needed to predict regional soil carbon dynamics.

The Great Hing’an Mountains, which are located at the edge of the Eurasian continuous permafrost region, has been considered to be ecosystem-driven or protected. The degradation of permafrost as reflected in increased ground temperature, deepening of the active layer, and fragmentation of the spatial distribution has been widely detected in the Great Hing’an Mountains (Jin et al., 2007). The microbial community succession profoundly affects soil carbon and nitrogen dynamics (Ward et al., 2013). Understanding how soil microbes regulate carbon emissions in the wetland-forest ecotone is significant for predicting the responses of soil ecosystems to degrading permafrost.

In this study, we conducted a 45-day incubation experiment using active-layer soils collected from the forest-wetland ecotone. The objectives of this study were: (1) to reveal the response of SOC mineralization and soil bacterial community to warming; and (2) to explore the environmental controls on SOC mineralization in permafrost area. We hypothesized that: (1) elevated temperatures would significantly stimulate the amount of SOC mineralization; (2) bacterial community composition varies with the incubation temperature and soil layer depth.

## Materials and Methods

### Site Description and Sampling

The study area (Supplementary Figure 1) was located in the continuous permafrost region of the northern slope of the Great Hing’an Mountains, Heilongjiang Province, China (53°28′N, 122°20′E). This area is in the cold temperate climate zone, with a mean annual temperature of −2.19°C, and a mean annual precipitation of 549.9 mm, about two-thirds of which falls between June and August (Dong et al., 2021). Forests and wetlands are widespread in this region. Considering the ecological importance and sensitivity response to environmental change, we selected a forest-wetland ecotone as the research habitat, which is a transitional area with an active layer depth of 110–130 cm that contains elements of both forest and wetland ecosystems. *Larix gmelinii* is the dominant tree species, interspersed with *Salix integra*, *Betula ovalifolia* Ruprecht, and *Salix myrtilloides*. The herbaceous plants are dominated by *Eriophorum vaginatum and Carex schmidtii*. Temperature monitors revealed temperatures at 5 cm depth ranged from −11.73 to 16.25°C with a mean value of 0.43°C. Soils at 130 cm depth begin to freeze from bottom to up after thawing for decades at late August.

Three 10 m × 10 m plots, separated 20 m from each other, were selected randomly within the research area after the active layer completely thawed in 2020. In each plot, a soil core was collected after manually removing the litter and vegetation from the surface and cut at 0–20, 20–40, 40–60, 60–80, 80–100, and 100–120 cm of depth. Considering the high experimental costs, we mix thoroughly the soils at same depth from different plots into a composite sample. Part of each sample was air-dried, crushed, and sieved to 0.25 mm for the initial physicochemical analysis. The remainder were sieved through a 4 mm mesh and homogenized for the incubation experiment.

### Sample Analysis and Incubation Experiment

The SOC and DOC contents of all soil samples were measured using a Multi N/C 3100 analyzer (Jena, Germany). The contents of soil nitrate nitrogen (NO_3_^–^-N) and ammonium nitrogen (NH_4_^+^-N) were analyzed using a SAN++ flow injection auto-analyzer (Skalar, Netherlands). Soil pH was measured in a 1:5 (m/v) soil-water slurry with a combination electrode (PHSJ-3F pH, Shanghai, China). The soil properties are summarized in Supplementary Table 1.

Because of the good drainage and aeration of sampling points at the end of growing season, we used aerobic C release as CO_2_ – C in this study. Considering that unrealistic warming may not effectively reflect the response of microorganisms and carbon mineralization. Therefore, we simulated lower and higher magnitudes of warming according to the actual temperature of the plot. Individual soil samples (equal to 50 g dry weight) maintained at field moisture conditions were incubated in 500 mL airtight glass jars sealed with a rubber stopper at 5°C (close to the average temperature of active layer at time of collection, 4.05°C) and 15°C (close to the annual maximum temperature of active layer, 16.25°C) in incubators (Thermo Fisher Scientific, United States). A total of 42 (2 temperature gradients × (6 soil depths + 1 empty) × 3 replications) bottles containing soil samples were prepared for the experiment. In order to eliminate the effects of transportation and homogenization on microorganisms as much as possible, we carried out pre-incubation for 7 days before measurements (Chen et al., 2016; Qin et al., 2021; Schroeder et al., 2021). Before sampling, the soil jars were flushed with air for 15 min to eliminate the previously accumulated gas, sealed with rubber plug for 2 h (Wang et al., 2014). A 15 ml volume of the gas sample was drawn using a 50 mL syringe equipped with a three-way valve. After sampling, the flasks were left open to replenish with fresh air. CO_2_ concentrations were measured by gas chromatography (Agilent 7890B, United States) on days 1, 3, 6, 10, 15, 21, 28, 36, and 45. The soil sample moisture content was kept stable by dropping deionized water for a weight balance after gas sampling.

The temperature sensitivity coefficient (*Q*_10_) is an index of temperature dependence, which represents the proportional change in rate given a 10°C change in temperature (Kirschbaum, 1995). It is a key indicator used to explore SOC decomposition under global warming (Conant et al., 2010; Suseela and Dukes, 2013; Nottingham et al., 2016). In this study, the *Q*_10_ value for carbon mineralization was calculated with the following equation:


$$Q_{10}={\left(\frac{K_{2}}{K_{1}}\right)}^{\frac{10}{T_{2}-T_{1}}}$$

Where *K*_1_ and *K*_2_ are the C mineralization rates (mg C kg^–1^ d^–1^) at 5°C (T1) and 15°C (T2), respectively.

### Microbiological Analysis

The microbial analysis was commissioned by Majorbio Bio-Pharm Technology Co., Ltd. (Shanghai, China). Genomic DNA of the microbial community was extracted from 0.3 to 0.5 g of fresh soil using the Soil DNA Kit (Omega Bio-Tek, United States), following the manufacturer’s instructions. The quality of the extract was determined by 1% agarose gel electrophoresis (Biowest agArose, United States), and DNA concentration and purity were determined using a NanoDrop 2000 UV-vis spectrophotometer (Thermo Fisher Scientific, United States). Universal primers (338F: ACTCCTACGGGAGGCAGCAG, 806R: GGACTACHVGGGTWTCTAAT) were used to amplify the 16S rRNA bacterial genes (95°C for 3 min, 27 cycles at 95°C for 30 s, 55°C for 30 s, and 72°C for 45 s, with a final extension of 72°C for 10 min and 10°C until termination). The polymerase chain reaction (PCR) reaction followed TransGen AP221-02 using 20 μL of TransStart Fastpfu DNA polymerase. Each sample was repeated three times and mixed to reduce the error in the DNA extraction process. The PCR products were purified using the AxyPrep DNA Gel Extraction Kit (Axygen Biosciences, United States) and quantified using QuantiFluor™-ST (Promega, United States). The amplicons were sequenced with the Illumina MiSeq Platform (Illumina Inc., United States) using the paired-ends model (PE300).

The raw 16S rRNA gene sequencing reads were demultiplexed, quality-filtered with fastp (version 0.20.0) (Chen et al., 2018), and merged using FLASH (version 1.2.7) (Mago and Salzberg, 2011). Operational taxonomic units (OTUs) with a 97% similarity cut-off were clustered using UPARSE (version 7.1) (Stackebrandt and Goebel, 1994; Edgar, 2013), and chimeric sequences were identified and removed. The taxonomy of each representative OTU sequence was analyzed against the 16S rRNA database (Silva v138-bacteria) using RDP Classifier (version 2.2) (Wang et al., 2007) with confidence values >0.7. To ensure that samples were compared at the same level, the OTU table was rarefied to 26,839 reads per sample prior to analyses. This value was the lowest sequencing depth obtained from a sample.

### Statistical Analysis

The differences in the soil physicochemical parameters, SOC mineralization and *Q*_10_ value were compared using one-way analysis of variance (ANOVA). Two-way ANOVA was used to analyze the effects of incubation temperature (T), soil layer (L), and their interactions (T × L) on SOC mineralization and the bacterial community structure. Stepwise regression analyses (SRAs) were used to reveal the main bacterial taxa controlling SOC mineralization. Above statistical analyses were implemented in SPSS 20.0 software (SPSS Inc., Chicago, IL, United States). Redundancy analysis (RDA) was conducted using CANOCO software 5.0 to determine the relationships between soil bacterial community and the physicochemical parameters. Non-metric multi-dimensional scaling (NMDS) and partial least squares discriminant analysis (PLS-DA) were conducted to visualize the differences of species diversity and classification based on the free online platform of Majorbio Cloud Platform. The Student’s *T*-test was used to identify differences in relative abundance and diversity indices between treatment groups. Figures were drawn using Origin Pro 2021 (Origin Software Inc., Northampton, MA, United States).

## Results

### Soil Organic Carbon Mineralization Responses to Warming

Cumulative SOC mineralization was higher at 15°C than at 5°C (Table 1). After the 45-day incubation, the greatest amount of SOC mineralization was 1,019.41 ± 141.23 mg CO_2_-C kg^–1^ soil C, which was observed in the 0–20 cm soil layer at 15°C. The lowest SOC mineralization was observed in the 20–40 cm soil layer at 5°C. The *Q*_10_ values of all soil layers ranged from 1.17 ± 0.30 to 3.84 ± 0.46 with an average of 2.17. And the values of 0–20 and 20–40 cm soil layer were significantly higher than other layers (Table 1).

**TABLE 1:** Amount of SOC mineralization and *Q*_10_ values of active layer soils.

Soil layer	SOC-mineralization (mg CO_2_-C kg^–1^ soil-C)		Q10
	5°C	15°C	
0–20 cm	351.57 ± 9.59^Ab^	1019.41 ± 141.23^Aa^	3.51 ± 0.88^A^
20–40 cm	240.10 ± 26.93^Bb^	745.74 ± 119.92^Ba^	3.84 ± 0.46^A^
40–60 cm	245.16 ± 20.70^ABb^	334.81 ± 14.24^Ca^	1.42 ± 0.12^B^
60–80 cm	280.99 ± 58.93^ABa^	335.12 ± 4.95^Ca^	1.17 ± 0.30^B^
80–100 cm	265.95 ± 32.55^ABb^	405.15 ± 42.89^Ca^	1.61 ± 0.46^B^
100–120 cm	254.50 ± 21.55^ABb^	351.74 ± 44.52^Ca^	1.50 ± 0.36^B^

*Values are means ± standard errors. Different capital letters indicate significant differences between the different soil layers at P < 0.05, and different lowercase letters indicate significant differences between the different treatments at P < 0.05.*

### Soil Bacterial Community Structure Responses to Warming

#### Changes in Bacterial Community Diversity

**FIGURE 1:**
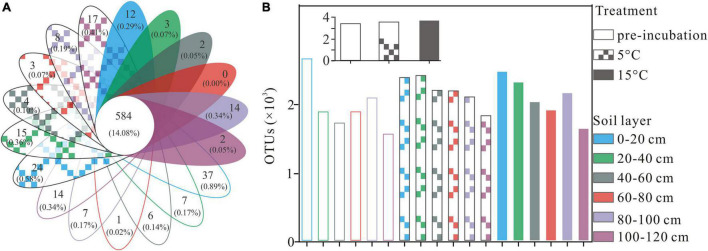
Venn diagram **(A)**, numbers of overlapping portions represent the number of OTUs that shared by all groups, and numbers of non-overlapping portions represent the number of unique OTUs for each sample, and column chart **(B)** represent the total number for each sample at OTU level, the small gray-colored figure in the top of subfigure **(B)** represent the mean OTUs of active layer soils at different treatment.

**FIGURE 2:**
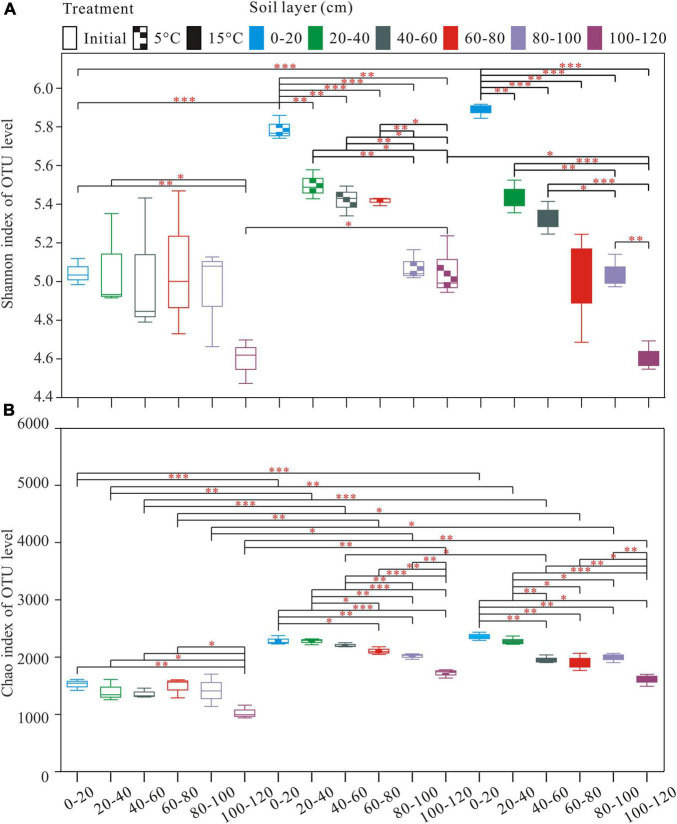
Alpha diversity Indices [Shannon **(A)**; and Chao **(B)**] index observed in the different treatments and depths. *P < 0.05, ^**^P < 0.01, ^***^P < 0.001.

A total of 2,990,739 optimized sequences were obtained and clustered into 4,148 OTUs. The Venn diagram analysis was carried out to facilitate comparison, which corresponds to 18 groups, including 3 temperature treatments and 6 soil layer subgroups. These 18 individual groups have different numbers of unique OTUs, with a total of 584 OTUs common to all groups (Figure 1A). The abundance of OTUs among soil layers ranged from 1,531 to 2,617, 1,791 to 2,376, and 1,608 to 2,431 in pre-incubated, 5°C, and 15°C-incubated soils, respectively, with the decreasing trend from the surface to the deep layer (Figure 1B). Among temperature treatments, the distribution of total OTUs followed the order 15°C (3,699) > 5°C (3,570) > pre-incubated (3411). Warming had a significant impact on the bacterial alpha-diversity of the active layer soils (Figure 2). In general, alpha diversity was higher in upper layer than in deeper layer soils. Shannon index rose strongly with a more pronounced increase in 0–20 cm layer than in other layers. Incubation temperature, soil layer and their interaction have significant effects on the Chao index (Supplementary Table 2).

**FIGURE 3:**
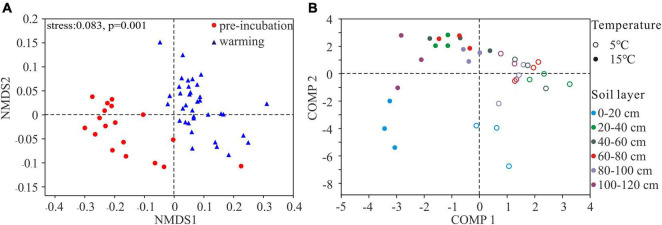
Bacterial community composition indicated by NMDS plot **(A)** and PLS-DA score plot **(B)**.

NMDS plots showed that the microbial community structures of pre-incubated soils clustered together but shifted strongly at 5 and 15°C (Figure 3A). Then, using PLS-DA to clarify the effects of incubating temperatures on bacterial communities (Figure 3B). The results indicated that bacterial communities at 5 and 15°C clustered separately, suggesting the overall structures of the bacterial communities under different temperature were clearly distinguished. Importantly, comp 1 could well separate samples at two temperatures, and comp 2 could almost separate the 0–20 cm top soil from the rest of active layer.

#### Comparison of Soil Bacterial Communities Among Samples

**FIGURE 4:**
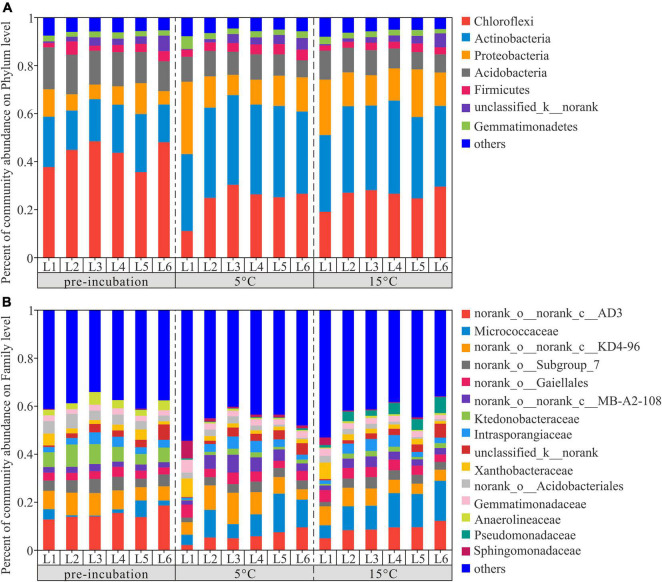
Distribution of bacterial community at phylum **(A)** and family **(B)** level across active layer soils under 3 temperature treatment. L1,…, and L6 are soil depths of 0–20,…, and 100–120 cm.

**FIGURE 5:**
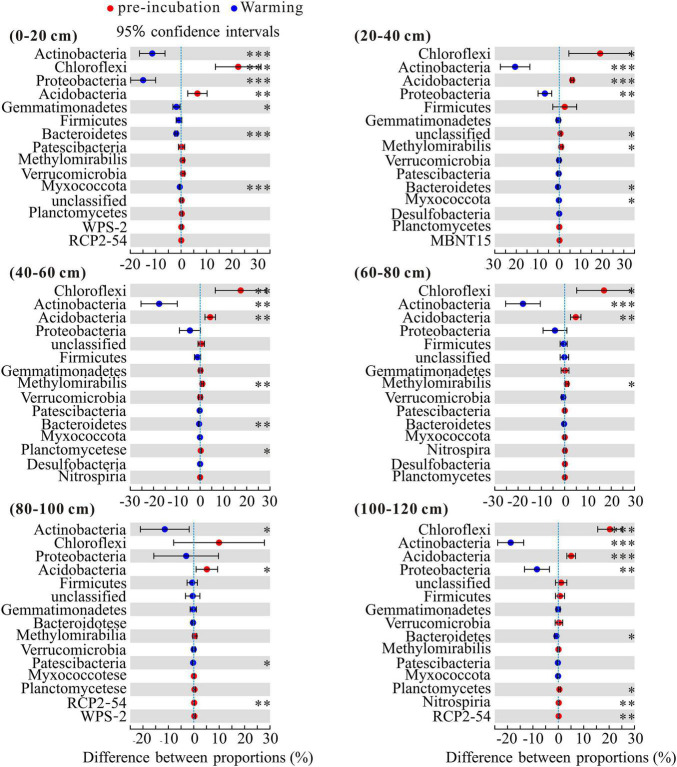
Statistical comparison of the relative abundance of the top 15 phyla between pre-incubation and warming treatment. Data of were showed as difference between proportions. Statistical analysis was evaluated by one-way ANOVA. *P < 0.05; ^**^P < 0.01; ^***^P < 0.001.

To investigate the changes in the bacterial community composition of soil samples, we compared the relative abundances at the phylum and family levels (Figure 4). Chloroflexi, Actinobacteria, Proteobacteria, and Acidobacteria were the dominant phyla across 3 temperature treatments among soil layers and together accounted for more than 80% of the community abundance, aside from unclassified bacteria (Figure 4A). Chloroflexi were the most abundant bacterial taxa of pre-incubated samples, with relative abundances of 37.66, 44.81, 48.39, 43.60, 35.54, and 48.01% in 0–20, 20–40, 40–60, 60–80, 80–100, and 100–120 cm, respectively; Acidobacteria were the most abundant bacterial taxa of incubated samples, and accounted for 32.03–38.01% and 31.94–38.78% of the community abundance in samples after 45-day incubation at 5 and 15°C, respectively. As shown in Supplementary Figure 2, the composition of soil dominant phyla at 5°C and 15°C were more similar, but different from that of pre-incubated soils. Two-tailed Student’s *t*-test showed the effect of warming on bacterial relative abundance varied among the soil layers and bacterial species (Figure 5). Warming statistically increased the relative abundance of Actinobacteriota across 6 soil layers, while decreased the relative abundance of Acidobacteriota, compared to pre-incubated soils. We further compared the relative abundance at phylum level between 5 and 15°C groups and found the major differences were pronounced in the 0–20 cm and 100–120 cm soil layers (Supplementary Figure 3).

The percentage of community abundance at the family level is shown in Figure 2B. The four dominant bacteria of active layer soils across three treatments were norank_o__norank_c__AD3, Micrococcaccac, norank_o__norank_c__KD4-96 and Ktedonobacteraceae, with relative abundances between 5.77–15.02%, 2.75–11.97%, 6.10–8.27%, and 1.50–6.56%, respectively (Supplementary Figure 4). The relative abundance of norank_o__norank_c__AD3 and Ktedonobacteraceae decreased significantly with the increase of temperature, while the abundance of Micrococcaccac increased. The statistical comparison of the relative abundance of the most abundant 15 bacterial species at the family level were depicted in Supplementary Figure 5. The results showed that there were 6, 2, 5, 4, 1, and 9 statistically significant differences among the pre-incubation, 5 and 15°C of 0–20, 20–40, 40–60, 60–80, 80–100, and 100–120 cm soil layers.

### Relationship Among Soil Parameters, Bacterial Communities, and Soil Organic Carbon Mineralization

**FIGURE 6:**
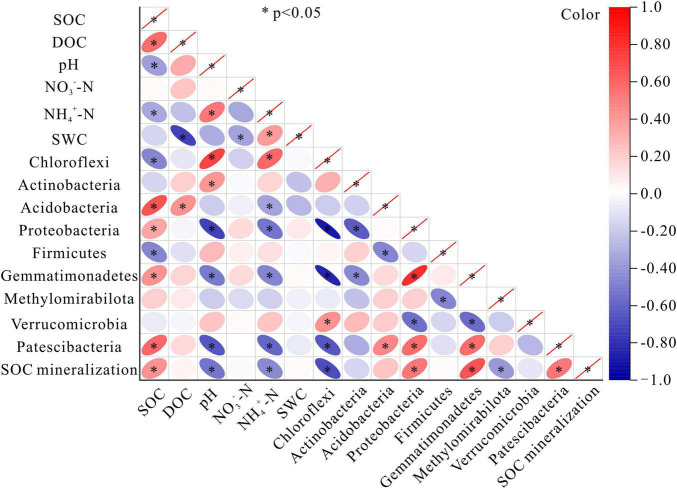
Correlation matrices of SOC mineralization for bacteria and the soil parameters. Colors indicated the direction of the correlation, where red, positive correlation; and blue, negative correlation. SOC, soil organic carbon; DOC, dissolved organic carbon; NO_3_^–^-N, nitrate nitrogen; NH_4_^+^-N, ammonium nitrogen; SWC, soil water content. * indicates a significant correlation at the 0.05 level.

The relative abundances of most of the bacterial phyla (except Actinobacteriota, Methylomirabilota, and Verrucomicrobiota) were correlated with SOC content. The relative abundance of Acidobacteria, Proteobacteria, Gemmatimonadetes, and Patescibacteria were all correlated positively with SOC content. In contrast, the relative abundance of Chloroflexi and Firmicutes were negatively correlated with SOC content (*P* < 0.05) (Figure 6). There was a noticeable absence of significant relationships between NO_3_^–^-N content or soil water content and the relative abundance of bacterial phyla. The positive correlations between pH and the bacterial phyla were seen in Chloroflexi, and Actinobacteriota, while the negative correlations were found in Proteobacteria, Gemmatimonadota, and Patescibacteria. DOC content was positively correlated with Acidobacteriota. Positive correlations were observed between NH_4_^+^-N content and Chloroflexi, while negative correlations were detected between NH_4_^+^-N content and Acidobacteriota, Proteobacteria, Gemmatimonadota, and Patescibacteria (Figure 6). The amount of accumulated SOC mineralization after the 45-day incubation was positively correlated with SOC content, but negatively correlated with pH and NH_4_^+^-N content (*P* < 0.05). The relative abundances of 5 of 9 bacterial phyla were significantly correlated with the amount of SOC mineralization, among which Chloroflexi and Methylomirabilota were negatively correlated, while Proteobacteria, Gemmatimonadota, and Patescibacteria were positively correlated (Figure 6).

**FIGURE 7:**
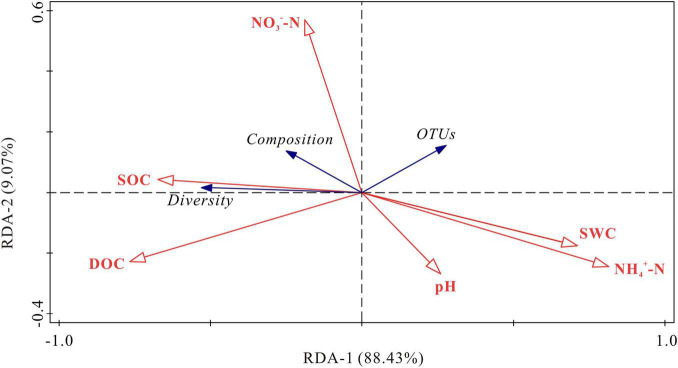
RDA for the relationship between soil bacterial community and physicochemical parameters. SOC, soil organic carbon; DOC, dissolved organic carbon; NO_3_^–^-N, nitrate nitrogen; NH_4_^+^-N, ammonium nitrogen; SWC, soil water content; Diversity, the Chao index; Composition, NMDS; OTUs, operational taxonomic units.

RDA demonstrated that the first two axis components explained 97.50% of the variance in the relationship between soil bacterial activities and the physicochemical parameters (Figure 7). Soil NH_4_^+^-N and DOC contents were the most important variables, which explained 59.4% (*P* < 0.01) and 25.9% (*P* < 0.05) of the variation in bacterial activities, respectively. The bacterial diversity (Chao index) was positively correlated with SOC content, and negatively correlated with NH_4_^+^-N content and soil water content (*P* < 0.05). The OTUs was negatively correlated with DOC content. Bacterial composition (NMDS 1) was positively correlated with SOC content and negatively correlated with pH, NH_4_^+^-N content and soil water content (*P* < 0.05). NO_3_^–^-N content had no significant correlations with OTUs, bacterial diversity (Chao index) and bacterial composition (NMDS 1) (*P* > 0.05). To remove multicollinearity, SRAs were established to quantitatively clarify the main soil bacterial species that controlled SOC mineralization in each warming treatment (Table 2). The results revealed that the effects of bacterial taxa on SOC mineralization at different temperatures were quite different. The bacterial phyla that associated with SOC mineralization at 5°C were Firmicutes and Proteobacteria, while the variation in SOC mineralization at 15°C was explained by Chloroflexi, Patescibacteria, Firmicutes, and Methylomirabilota.

**TABLE 2:** Stepwise regression analyses of the soil bacterial composition and SOC mineralization.

Temperature	Adjusted *R*^2^	*F*-statistic	*P*-value	Significant variables (*P* < 0.05)	Beta coefficient
5°C	0.591	11.489	0.004	Firmicutes	–0.684
				Proteobacteria	0.533
15°C	0.917	11.278	0.005	Chloroflexi	–0.584
				Patescibacteria	0.403
				Firmicutes	–0.274
				Methylomirabilota	–0.246

## Discussion

### Soil Organic Carbon Mineralization and Temperature Sensitivity

Soil organic carbon mineralization and its temperature sensitivity have become the main focus of soil carbon dynamic studies in the context of global warming (Wagai et al., 2013). In general, increasing temperature within an appropriate range significantly improves SOC mineralization (Davidson and Janssens, 2006; Wang et al., 2010, 2014; Song et al., 2014; Jiang et al., 2020). The same result was found in the present incubation when the temperature treatment was increased from 5 to 15°C. However, we found that the amount of SOC mineralization related to peatlands in the same region were approximately 2–3 times higher than the values of our study (Song et al., 2021), which may be attributed to the lower amount of organic carbon (Supplementary Table 1). It is generally believed that peatlands are important carbon pools in permafrost regions (Turunen et al., 2002; Hugelius et al., 2014; Song et al., 2018). The SOC content in this study were 1.5–2.8 times lower than the values of peatlands in the same area reported by Jiang et al. (2020) and Song et al. (2021). Whereas the changes of habitat suitability may influence microbial community and then affect the decomposition of organic matter, resulting in changes in greenhouse gas emission (Jorgenson et al., 2001; Inglett et al., 2012; Ward et al., 2013). Consistent with Jiang et al. (2020), the amount of SOC mineralization in the shallow soil layers (0–20 and 20–40 cm) were higher than those in the deeper layers (Table 1). This phenomenon could be easily explained by the microbial response to warming and the content of organic carbon. It can be seen from Table 1 that upper layers have higher organic carbon, which is considered an important substrate for microorganisms. The variations in the soil microorganisms are likely to have profound impacts on soil nutrient cycling. Yergeau et al. (2012) found that warming can stimulate soil respiration by changing microbial community composition and abundance. Consistent with expectations, warming enhanced bacterial diversity and widened its difference between upper layers and other layers in this study. Thereby, we can safely conclude that the upper layers have a stronger CO_2_ release potential than the deeper layers.

The *Q*_10_ value and its influencing factors differ between various ecosystems. Previous studies have demonstrated that higher variability in the *Q*_10_ value is related to soil properties, microbial community, incubation conditions, and calculation method (Haddix et al., 2007; Hartley and Ineson, 2008; Craine et al., 2010; Hamdi et al., 2013; Karhu et al., 2014; Lin et al., 2015). The average *Q*_10_ value of SOC mineralization for six soil layers of active layer was 2.17 (range of 1.17–3.84) (Table 1). The *Q*_10_ values of 0–20 cm was higher than the global average *Q*_10_ value (2.41) (Wang et al., 2019) and close to the values (*Q*_10_ = 2.24–4.22) measured by Song et al. (2021) in peatlands of the same region. Based on the analysis above, the least that can be concluded is that forest-wetland ecotone has lower organic carbon content and higher temperature sensitivity compared with peatlands. It is widely known that soil substrate quality and quantity control the decomposition of SOC by microorganisms (Jagadamma et al., 2014). Therefore, we confirmed that SOC mineralization from the forest-wetland ecotone of the permafrost region in the Great Hing’an Mountains is sensitive to increases in temperature.

### Soil Bacterial Community Structure

Chloroflexi, Actinobacteria, Proteobacteria, and Acidobacteria were the most dominant phyla in this study. These phyla have also been documented in soils under various environment by other researchers (Wang et al., 2017; Luláková et al., 2019; Li et al., 2020), suggesting these phyla have a wide ecological amplitude and superior adaptability. The effect of warming on bacterial abundance varied among the soil layers, and the species acting on SOC mineralization at different temperatures were quite different. Changes in the relative abundance of bacterial species caused by warming were closely related to ecological strategies (Supramaniam et al., 2016). Elevated temperatures usually promote nutrient mineralization, which increase soil nutrient availability and provide more substrates for microorganisms. Although Acidobacteria were reported to have high substrate affinities, the proportion of Acidobacteria in warmed soils decreased, showing vulnerability of this phylum to warming (Riah-Anglet et al., 2015). Fierer et al. (2007) also found that the relative abundances of Acidobacteria decreased with increasing carbon mineralization accentuating the predictive function of carbon mineralization for the relative abundances of Acidobacteria. Consistent with our findings, higher relative abundance of Actinobacteria were also found in other soil-warming studies (DeAngelis et al., 2015; Wu et al., 2015; Chong et al., 2018). Soil warming would significantly increase the amount of carbohydrates (Pold et al., 2016). Habtewold et al. (2021) suggested that warming may stimulate bacteria with higher growth rates and resource utilization efficiency. The phylum Actinobacteria were considered to contain many representative taxa known to degrade recalcitrant carbon (Barret et al., 2011). Moreover, the genomes of Actinobacteria were generally enriched in glycoside hydrolases responsible for degrading cellulose, starch, and xylan, compared to other phyla (Pold et al., 2016). Therefore, the increase of labile substrates caused by warming might enrich potentially copiotroph communities resulting in significant implications on future soil carbon cycle processes with warming.

Although the relative abundances of certain bacteria in all layer soils did respond to warming, the most pronounced changes occurred in upper layer soils (Figure 5). In line with our results, Ricketts et al. (2020) also found that the soil layer was a primary explanatory variable and driver of bacterial community structure. This is due to the resource availability and redox potential of bacterial community were determined by inherent microscale changes in soil matrix. In addition, Yergeau et al. (2009) pointed out the response of microorganisms to environmental change are closely related to enzymes. And our previous studies showed that upper layers exhibited higher β-glucosidase and phosphatase activity (Liu et al., 2021). The presence of these enzymes suggested rich environment of C sources (Coolen et al., 2011). Actinobacteria and Proteobacteria are considered be positively correlated with available carbon and can grow rapidly in an environment rich in available carbon (Fierer et al., 2007; Schostag et al., 2019). In contrast, the relative abundance of oligotrophic Acidobacteria (Ho et al., 2017) in these soils generally decreased in response to warming (Figure 4).

Warming significantly increased the bacterial alpha-diversity, independent of the soil layers, and the results of the NMDS plots showed that there were obvious differences in soil bacterial composition between pre-incubated and warming soils (Figure 3A), which were consistent with other studies in cold regions (Xiong et al., 2014; Wu et al., 2015; Luláková et al., 2019; Yu et al., 2019). We speculated that elevated temperature makes carbon sources more accessible (Hartley et al., 2008), leading to community structure transformation through biochemical adaptations with increased diversity (Allison and Martiny, 2008). PLS-DA results showed that two sample groups treated at 5 and 15°C have obvious dispersion (Figure 3B), indicating the adjustment of community composition and structure may be an important response to change in temperature. Luo et al. (2001) reported that thermal adaptation of microbial processes may change with microbial community structure. Thermal adaptation theory emphasizes the adaptability of bacterial growth under extreme temperatures (Davidson and Janssens, 2006; Schindlbacher et al., 2011; Streit et al., 2014).

### Linking Soil Organic Carbon Mineralization and the Soil Parameters With the Soil Bacterial Community

Soil organic carbon may be more easily decomposed by microorganisms in the case of climate warming, potentially causing positive carbon feedback to climate change (Schuur et al., 2015; Liu et al., 2019). This decomposition process is regulated by a set of interacting systematically organized factors (Carrillo et al., 2012; Xu et al., 2021) that largely depend on soil hydrothermal conditions, soil substrates, and the activities of decomposers. Nevertheless, the information of microbial responses to warming and how they link to SOC mineralization in alpine ecosystem remains largely elusive. We observed significant interactive effects (*P* < 0.05) between the soil layers and incubation temperatures on cumulative SOC mineralization, bacterial diversity and community composition (Supplementary Table 2). Despite we firmly believed that soil microbial decomposition under global warming may potentially change SOC storage in the permafrost region, the mechanism of SOC mineralization and microbes in the active layers in response to warming is difficult to understand. Ernakovich et al. (2017) showed that microbial community structure and DOC content in thawed permafrost are good predictors of CO_2_ emissions. Zhu et al. (2021) found that microbial composition was closely related to soil carbon content in wetland. Our results showed that SOC mineralization was positively correlated with SOC content, as well as with the relative abundances of most bacterial phyla and diversity (*P* < 0.05). Moreover, SOC content was positively correlated with the relative abundance of most soil bacterial phyla and diversity. Soil NH_4_^+^-N and DOC contents were the main variables explaining bacterial activities. These results were consistent with findings from the same area, which emphasized the importance of available carbon in regulating the response of soil carbon to warming (Song et al., 2021). Rousk et al. (2012) found that the temperature adaptation for bacterial growth to higher temperatures is overshadowed by the direct temperature effect on substrate depletion. Song et al. (2018) reported that elevated temperature accelerates the decomposition of the available soil carbon component in a short time and reduces the supply of the substrate during the later period of incubation. You et al. (2021) assessed the effects of exogenous carbon addition on SOC mineralization and its temperature sensitivity through a 42-days incubation and pointed out that once the labile soil carbon is depleted by mineralization, the added carbon may promote the decomposition. Therefore, we suggest that the content of carbon substrates is the main driving force of SOC mineralization under warming condition.

## Conclusion

We conducted a 45-day incubation experiment using active layer soils collected from the forest-wetland ecotone in the Great Hing’an Mountains of Northeast China to determine how soil organic carbon mineralization and bacterial communities respond to increased temperatures. Our study demonstrated that elevated temperatures significantly increased SOC mineralization across the active layer soils in forest-wetland ecotone. Warming had great promotion on the bacterial alpha diversity and could separately cluster the bacterial communities. The percentage of community abundance at the family level varied among the soil layers and temperature treatment, but the dominant phyla did not change with increasing temperature. The relationship among SOC mineralization, soil parameters and bacterial community showed microbial regulation of SOC mineralization was strongly mediated by the availability of carbon substrates. Considering the continuous warming, further studies should measure microbiological community and available substrate at different stages to enhance our understanding of carbon release under permafrost degradation.

## Data Availability Statement

The original contributions presented in the study are included in the article/Supplementary Material, further inquiries can be directed to the corresponding author/s.

## Author Contributions

XD: conceptualization and writing – original draft. CL: formal analysis and review and editing. DM and XW: methodology. YW and HM: investigation. ML: review and editing, and funding acquisition. SZ: project administration and funding acquisition. All authors contributed to the article and approved the submitted version.

## Conflict of Interest

The authors declare that the research was conducted in the absence of any commercial or financial relationships that could be construed as a potential conflict of interest.

## Publisher’s Note

All claims expressed in this article are solely those of the authors and do not necessarily represent those of their affiliated organizations, or those of the publisher, the editors and the reviewers. Any product that may be evaluated in this article, or claim that may be made by its manufacturer, is not guaranteed or endorsed by the publisher.
