# Supplementary material for: Organic Carbon Mineralization and Bacterial Community of Active Layer Soils Response to Short-Term Warming in the Great Hing’an Mountains of Northeast China
Source: Front Microbiol. 2021 Dec 24;12:802213. doi: 10.3389/fmicb.2021.802213 (PMC8739994; doi:10.3389/fmicb.2021.802213)
Supplement: Supplementary file 1 [file Data_Sheet_1.docx]

***Supplementary Material***

1. **Supplementary Figures and Tables**
   1. **Supplementary Figures**


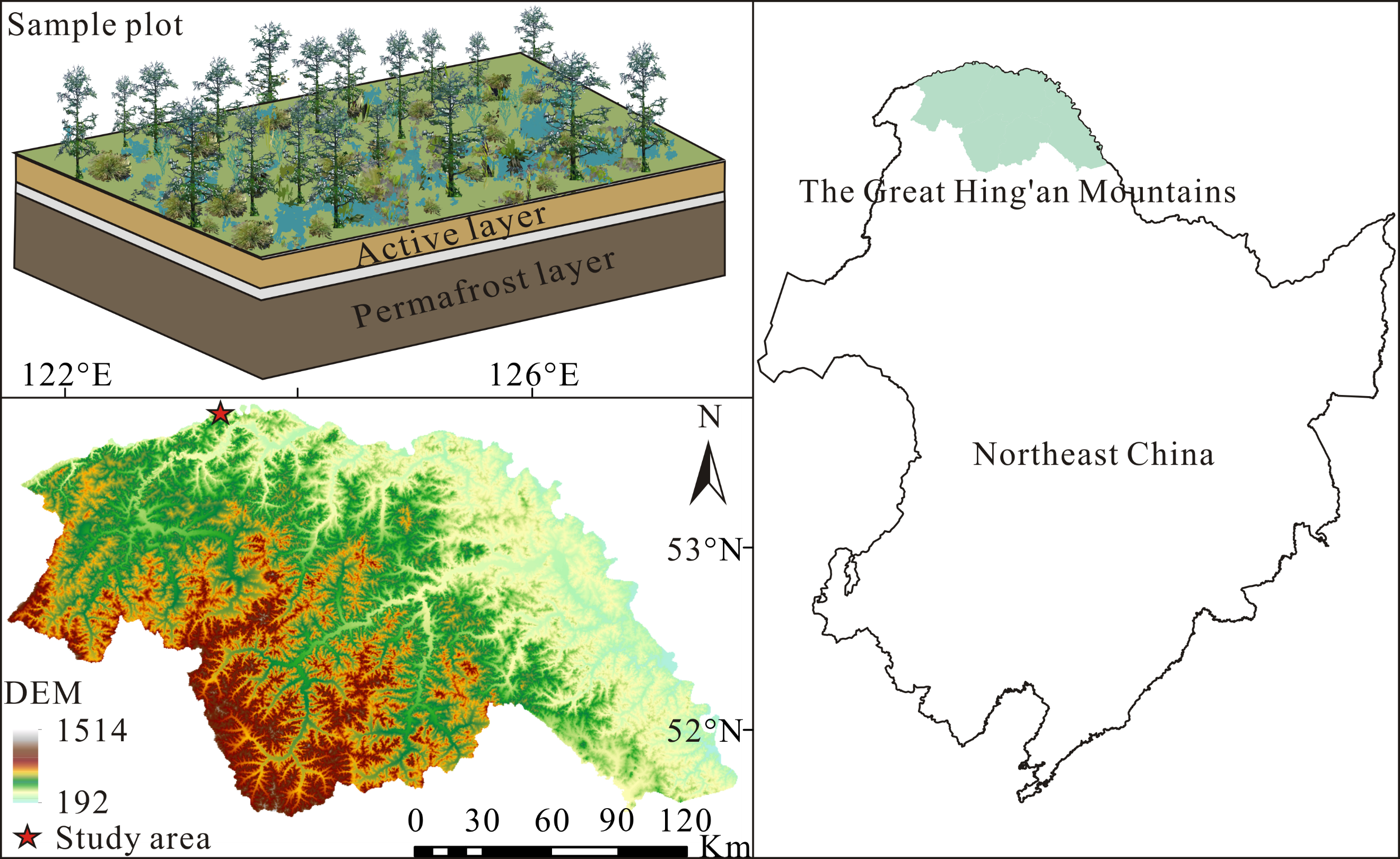


**Supplementary Figure 1.** Location of the sampling site in the Great Hing'an Mountains, northeastern China.


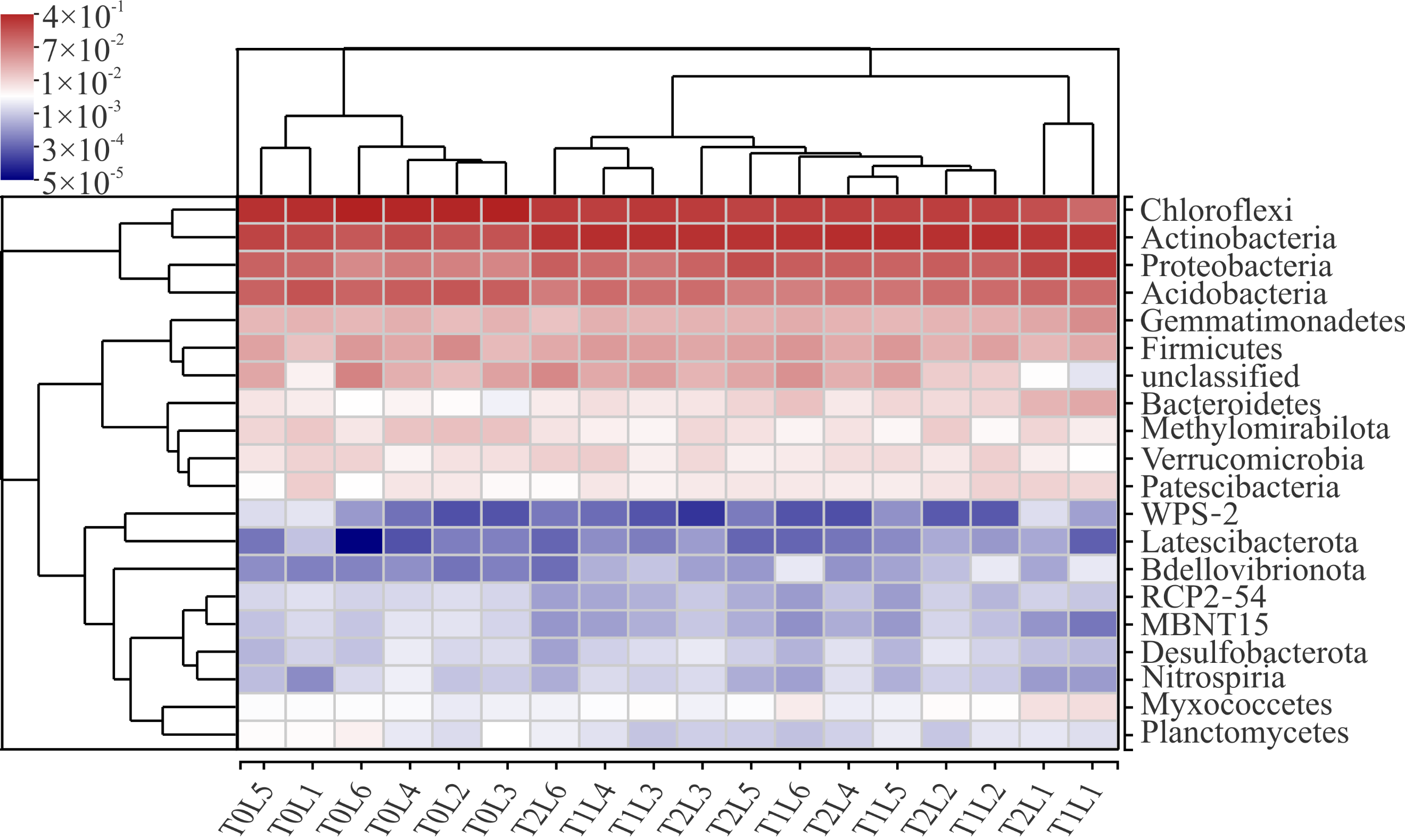


**Supplementary Figure 2.** Heat map showed the relative abundance of bacterial phyla of active layer soils. T0, T1 and T2 represent pre-incubation, 5°C and 15°C, respectively. L1 to L6 refer to 0-20 cm to 100-120 cm. The abundance changes of different species in the sample were shown by the gradient (abundance logarithm) of color patches.

**
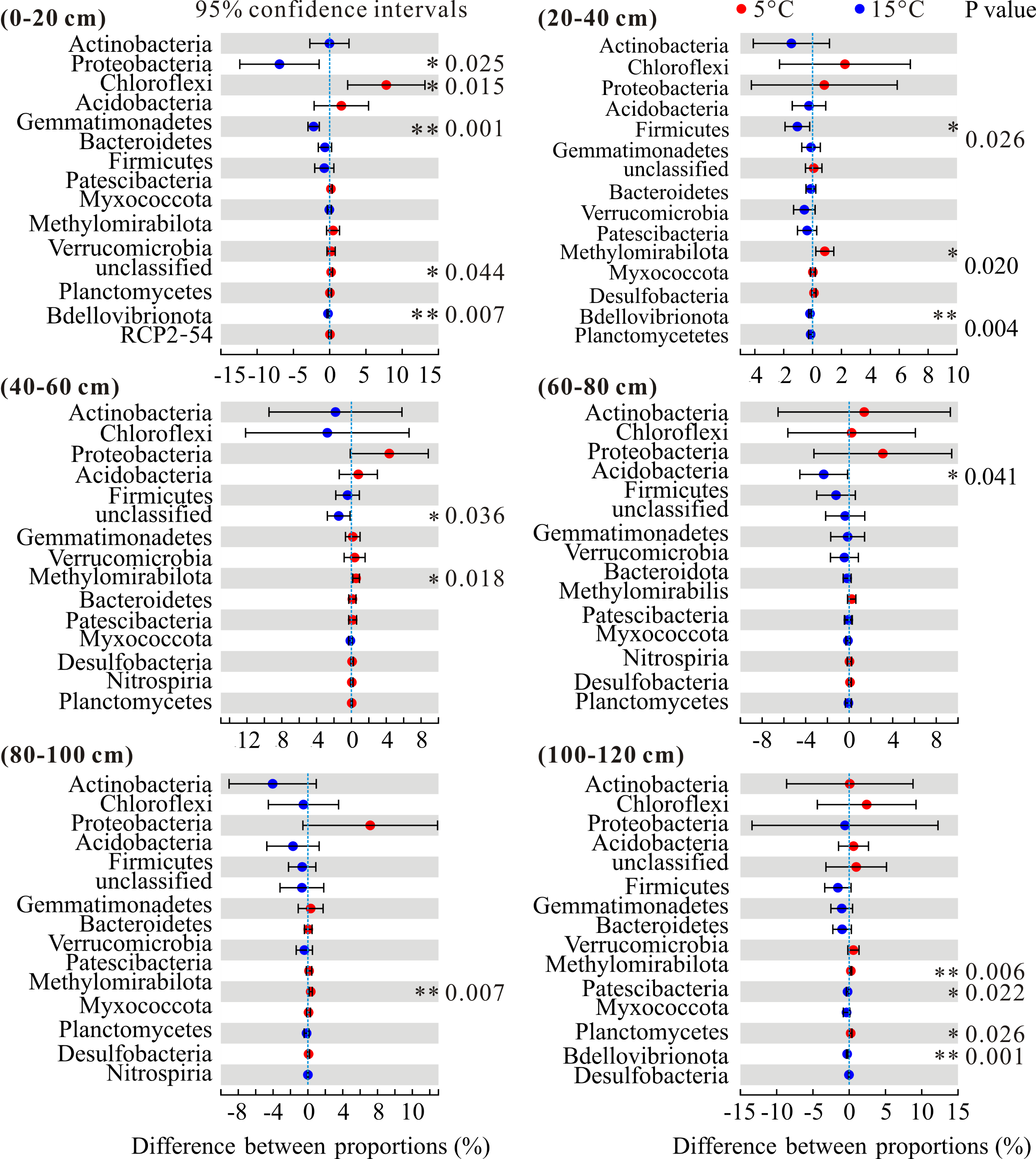
 Supplementary Figure 3.** Extended error bar plot showing the differences in relative abundance of the top 15 phyla between 5°C and 15°C incubation. Data of were showed as difference between proportions. Statistical analysis was evaluated by Student’s t-test. *P<0.05; **P<0.01; ***P<0.001.

**
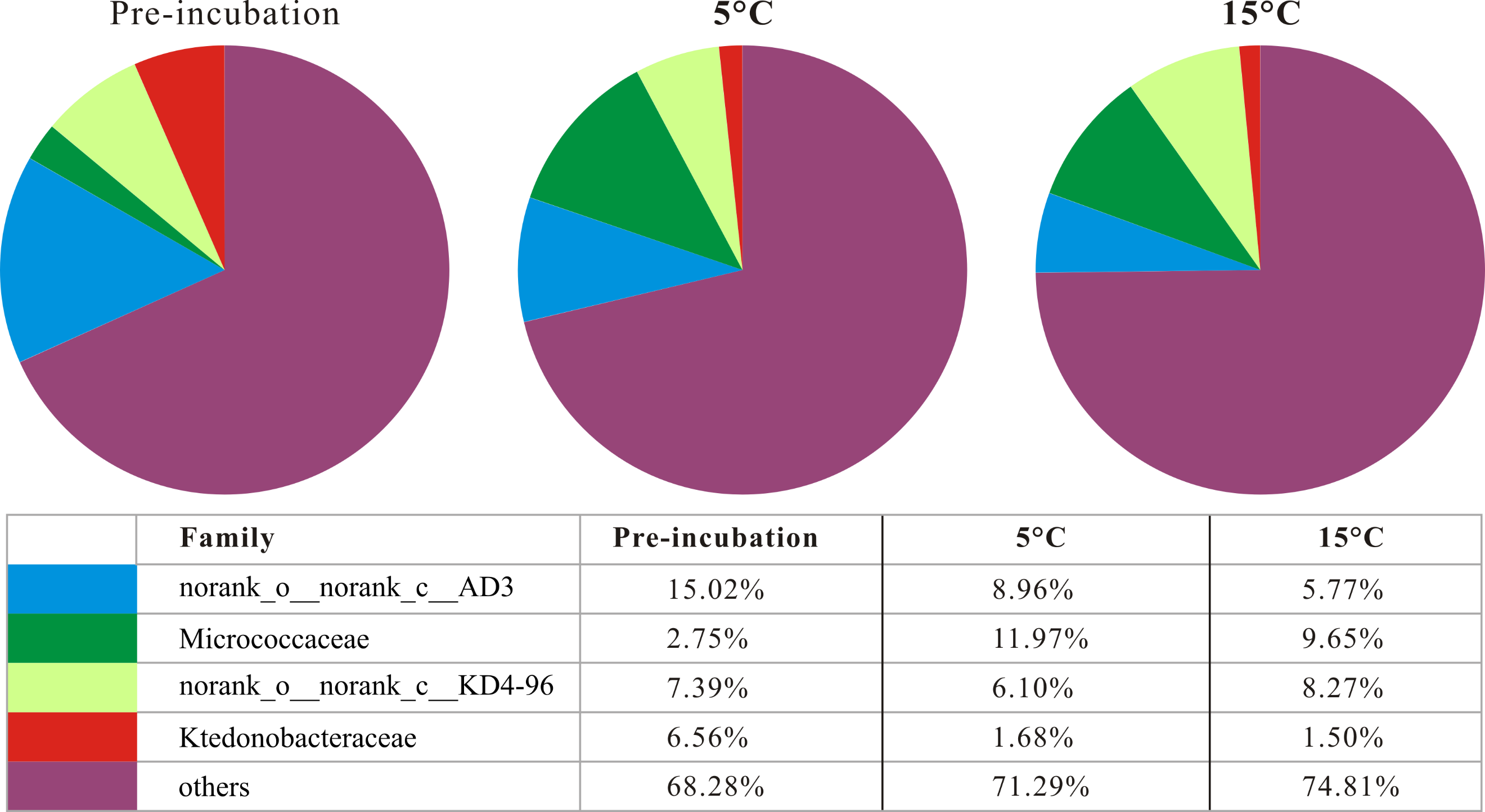
**

**Supplementary Figure 4.** Bacterial distribution of the dominant families of active layer soils under different temperature treatment at the family level. Pie charts show the proportion of reads.

**
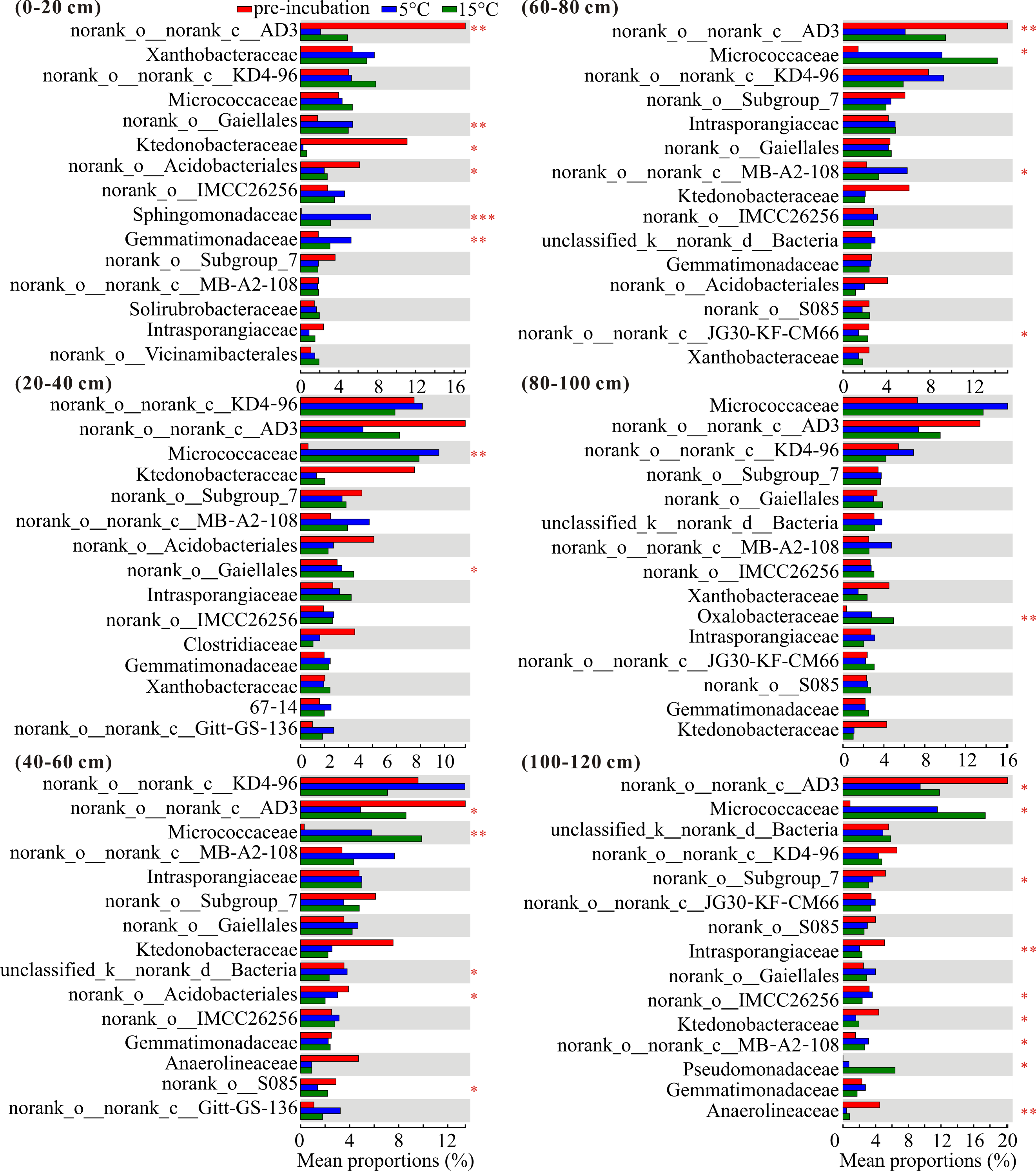
**

**Supplementary Figure 5.** Statistical comparison of the relative abundance of the top 15 families among 3 temperature treatment. Data of were showed as difference between proportions. Statistical analysis was evaluated by one-way ANOVA. *P<0.05; **P<0.01; ***P<0.001.

## Supplementary Tables

## Supplementary Table 1. Initial soil properties. Values are means ± standard errors. Different capital letters indicate significant differences between the different soil layers at P < 0.05. SOC, soil organic carbon; DOC, dissolved organic carbon; NO_3_^-^-N, nitrate nitrogen; NH_4_^+^-N, ammonium nitrogen; SWC, soil water content.

| Soil layer  (cm) | SOC  (g kg^-1^) | DOC  (mg kg^-1^) | NH_4_^+^-N  (mg kg^-1^) | NO_3_^-^-N  (mg kg^-1^) | pH | Soil water content  (%) |
| --- | --- | --- | --- | --- | --- | --- |
| 0-20 | 117.77±1.90^A^ | 103.90±4.97^AB^ | 25.27±0.31^C^ | 8.15±3.21^A^ | 4.56±0.01^C^ | 23.73±0.14^B^ |
| 20-40 | 105.50±2.38^AB^ | 97.93±14.48^AB^ | 25.87±0.21^BC^ | 4.65±0.65^A^ | 4.77±0.13^B^ | 22.87±0.79^B^ |
| 40-60 | 98.03±3.24^BC^ | 113.50±1.49^AB^ | 27.33±0.52^AB^ | 6.34±1.00^A^ | 5.02±0.02^A^ | 20.11±0.77^B^ |
| 60-80 | 105.90±2.38^AB^ | 122.37±10.44^A^ | 27.33±1.03^AB^ | 9.55±3.14^A^ | 4.93±0.02^AB^ | 19.96±0.24^B^ |
| 80-100 | 92.21±5.58^C^ | 89.67±4.29^BC^ | 25.90±0.33^BC^ | 9.14±0.88^A^ | 4.81±0.03^B^ | 19.68±0.19^B^ |
| 100-120 | 93.75±1.63^C^ | 64.13±4.60^C^ | 28.53±0.45^A^ | 5.54±0.26^A^ | 4.8±0.14^B^ | 30.60±0.03^A^ |

**Supplementary Table 2.** Results of two-way ANOVA (*F* and *P* values) to test the effects of incubation temperature (T) and soil layer (L), and their interactions (T×L) on SOC mineralization, bacterial abundance (OTUs), diversity (Chao index) and composition (NMDS).

| Variations | T | | L | | T×L | |
| --- | --- | --- | --- | --- | --- | --- |
|  | *F* | *P* | *F* | *P* | *F* | *P* |
| SOC mineralization | 109.605 | <0.001 | 26.484 | <0.001 | 18.066 | <0.001 |
| Abundance | 0.879 | 0.358 | 0.667 | 0.652 | 2.092 | 0.101 |
| Diversity | 9.687 | 0.005 | 50.293 | <0.001 | 3.531 | 0.016 |
| Composition | 0.241 | 0.628 | 15.098 | <0.001 | 3.758 | 0.012 |
